# Drug Repurposing Campaigns for Human Cytomegalovirus Identify a Natural Compound Targeting the Immediate-Early 2 (IE2) Protein: A Comment on “The Natural Flavonoid Compound Deguelin Inhibits HCMV Lytic Replication within Fibroblasts”

**DOI:** 10.3390/v11020117

**Published:** 2019-01-29

**Authors:** Beatrice Mercorelli, Anna Luganini, Giorgio Palù, Giorgio Gribaudo, Arianna Loregian

**Affiliations:** 1 Department of Molecular Medicine, University of Padua, 35121 Padua, Italy; beatrice.mercorelli@unipd.it (B.M.); giorgio.palu@unipd.it (G.P.); 2 Department of Life Sciences and Systems Biology, University of Turin, 10123 Turin, Italy; anna.luganini@unito.it

Human cytomegalovirus (HCMV) is a ubiquitous herpesvirus that establishes a lifelong persistence in the host through both chronic and latent states of infection [1,2]. HCMV is a major opportunistic pathogen for immunocompromised individuals, such as transplant recipients—to whom the virus is a major cause of morbidity and mortality—and fetuses to which it is the leading viral cause of congenital defects [3]. Prevention and control of HCMV infections and diseases remain a major challenge. In fact, an effective vaccine is still lacking [4], and only a limited number of drugs, all targeting viral enzymes (i.e., DNA polymerase or terminase), are licensed to manage HCMV diseases [5]. There are still unmet medical needs regarding HCMV-associated infections, including prevention and therapy of congenital infection and management of drug-resistance, that call for the development of new, safe, and effective anti-HCMV compounds, possibly endowed with novel mechanisms of action. In this context, drug repurposing is an emerging strategy for antiviral drug discovery (reviewed in [6]). We and others have pursued such strategy to identify both new anti-HCMV compounds and novel targets of therapeutic intervention [7,8,9].

In this regard, three independent drug repurposing screenings directed against HCMV have been recently reported that used the same collection of small molecules (i.e., the Spectrum Collection from Microsource Discovery System, Inc.) [7,8,9]. The experimental approaches adopted in these screenings were basically two, either a phenotypic-based or a mechanism-based screening. In fact, in two cases, the screening assays were based on the evaluation of the expression of an indicator viral protein fused to the Enhanced Green Fluorescent Protein (EGFP), namely the Immediate Early 2 (IE2) in Gardner et al. [7], or the Late (L) UL99 in Nukui et al. [9], respectively. Expression of IE2-EGFP or UL99-EGFP in cells infected with the respective recombinant viruses was therefore used as a screening readout. In contrast, the screening by Mercorelli et al. [8] was a mechanism-based screening, designed specifically to identify compounds able to interfere with the transactivating activity of the prototypic viral transcription factor IE2. To this aim, a cell-based assay made up of an engineered human cell line stably expressing EGFP under the control of an IE2-inducible Early (E) promoter (i.e., the *UL54* gene promoter) was exploited [10]. Expression of EGFP upon infection of the indicator cell line with HCMV AD169 was therefore used as a screening readout. Thus, although all three screenings were based on a reporter gene expression analysis, the experimental design appears conceptually different. Other differences that can be seen among the three screenings concern the experimental setting, including target cell type, virus strains, compound doses, and the schedule of treatment. In this regard, it is known that in antiviral drug repurposing campaigns, even though the same library and the same pathogen are under investigation, different hits may be identified [6]. Nevertheless, in the case of the three mentioned screenings which targeted distinct but sequential steps of the HCMV gene expression cascade (Figure 1), one could expect that the same hits should have emerged from more than one screening. However, identical compounds were identified only by Mercorelli et al. [8] and Nukui et al. [9]. Among these, the natural compound deguelin was further characterized independently by both groups. 

Deguelin (DGN) is a flavonoid with specific pharmacological properties and is currently under preclinical investigation for its anti-cancer properties [11]. DGN was identified first by our group [8], then by Nukui et al. [9], as a specific and nontoxic inhibitor of HCMV replication. The broad-spectrum anti-HCMV activity of DGN was investigated by both groups: Nukui et al. [9] tested this compound against two bacterial artificial chromosome (BAC)-reconstituted viruses (UL99eGFP and TReGFP, derived from TB40/E and TR strains, respectively) [9], of which TReGFP is resistant to ganciclovir and cidofovir [12]. On the other hand, we tested DGN against several strains of HCMV, including laboratory strains, several strains resistant and cross-resistant to the available anti-HCMV drugs, as well as low-passaged clinical strains [8]. The different experimental setting (i.e., cell type, virus strains, multiplicity of infection, incubation time, type of assay, and readout) might explain the differences in the EC_50_ values measured for DGN in the two studies (EC_50_ = 1.3 ± 1.0 µM in [8] versus EC_50_ = 0.072 µM in [9]).

Nevertheless, in both studies, DGN showed inhibitory effects on both viral DNA synthesis and E and L viral gene expression. On the basis of these findings from both studies, it was hypothesized that DGN inhibits either a viral or a cellular function required for the switch from IE to E gene expression, thus blocking the essential viral E proteins expression required for replication of the HCMV genome.

To verify this hypothesis, we further investigated the mechanism of action of DGN against HCMV. Consistently with our initial experimental design, we observed that DGN, along with other compounds identified in our screening, interferes specifically with the transactivating activity of the viral transcription factor IE2, which is required for the productive activation of essential E gene promoters, such as *UL54* and *UL112-113* [8]. In fact, DGN treatment was able to significantly reduce viral IE2-dependent E gene expression in both HCMV-infected cells, and uninfected cells ectopically expressing IE2 in a reporter-based assay [8]. Interestingly, this mechanism of action was observed also for other hit compounds identified in our drug repurposing screen. In fact, the antiparasitic drug nitazoxanide, the antibiotic alexidine, thioguanosine (a metabolite of the anticancer drug thioguanine, which was also identified in the screening by Nukui et al. [9]), the calcium channel blocker manidipine, and the natural compound berberine, were all found to inhibit viral E gene expression by interfering with the transactivating activity of IE2 [8,13,14].

DGN possesses multi-faceted biological properties, being a multi-functional kinase inhibitor [11]. In fact, it inhibits several host cell signaling pathways that are conducive to HCMV replication, such as PI3K/Akt [15], mTOR [11], NF-κB, and VEGF signaling [16]. Thus, as envisaged by Nukui et al. [9], it is likely that DGN inhibits HCMV replication by interfering with the activation of one or more of these host factors whose functions contribute to the switch from IE to E phase of HCMV replication cycle and, in particular, to the IE2-dependent transcriptional activation. In this regard, however, we think that inhibition of the mTOR pathway is not likely the main mechanism underlying the anti-HCMV activity of DGN, since mTORC1 inhibition was reported to have no significant effects on HCMV replication, and IE and UL44 proteins expression [17]. To further sustain this view, it is worth noting that rapamycin, even though present in the compound library that we screened, was not selected by our IE2-dependent cell-based assay. Furthermore, we also consider unlikely that inhibition of cellular transcription factors that regulate the Major Immediate Early Promoter (MIEP) of HCMV (such as NF-κB, CREB, SP-1, AP1, ETS) is the primary mechanism of the antiviral activity of DGN. Indeed, an inhibitory effect on the activity of those transcription factors would lead to the inhibition of viral IE1 and IE2 protein expression which, on the contrary, was neither observed by us [8] nor by Nukui et al. [9]. However, since differences may exist in the regulation of MIEP between quiescent and proliferating cells [18], an inhibitory effect of DGN on some of these pathways, such as NF-κB, cannot be completely ruled out. Therefore, it is likely that other host factors might be involved in the antiviral mechanism of DGN and further studies are needed to elucidate the detailed molecular mechanisms. Nevertheless, the independent identification of DGN as a novel inhibitor of HCMV replication by two independent drug repurposing screenings, and its unique antiviral properties, fully justify its further development as an alternative anti-HCMV agent.

## Figures and Tables

**Figure 1:**
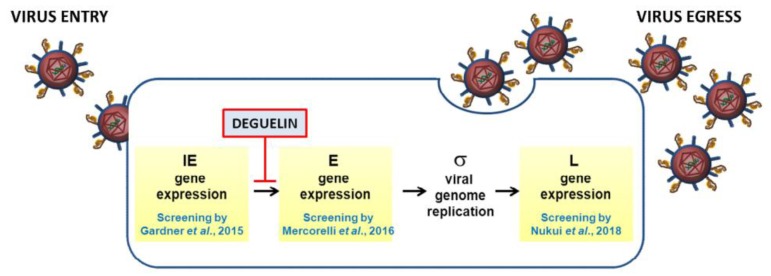
Different stages of the HCMV replication cycle targeted by drug repurposing campaigns. The schematic depicts the sequential steps of the HCMV gene expression cascade that have been targeted by the three indicated screening studies (IE, Immediate Early; E, Early; σ, sigma (stands for DNA replication by the rolling circle mechanism; L, Late). Deguelin is a flavonoid selected in the screenings by Mercorelli et al. [8] and Nukui et al. [9] and further characterized for its anti-cytomegaloviral activity and the ability to inhibit the expression of viral E genes.
